# Predicting early endodontic treatment failure following primary root canal treatment

**DOI:** 10.1186/s12903-024-03974-8

**Published:** 2024-03-12

**Authors:** Young-Eun Jang, Yemi Kim, Sin-Young Kim, Bom Sahn Kim

**Affiliations:** 1 Department of Conservative Dentistry, College of Medicine, Ewha Womans University, Seoul, South Korea; 2 Department of Conservative Dentistry, Seoul St. Mary’s Hospital, College of Medicine, The Catholic University of Korea, Seoul, South Korea; 3 Department of Nuclear Medicine, College of Medicine, Ewha Womans University, Seoul, South Korea

**Keywords:** Endodontic failure, Missed canals, Retreatment, Causative factors, Time to endodontic treatment failure

## Abstract

**Background:**

Understanding when and why endodontic treatments fail could help clinicians make prognoses and thus improve treatment outcomes. This study was aimed to assess potential predictors of early endodontic treatment failure. We explored factors contributing to the failure of initial root canal treatment were explored, with a specific emphasis on evaluating the influence of the time elapsed since the initial treatment.

**Methods:**

This retrospective cohort study enrolled 1262 patients who sought endodontic treatment at our department and 175 patients were included for analysis. Potential causes of endodontic treatment failure were investigated, such as inadequate obturation quality, inadequate coronal status, the presence of additional untreated canals, anatomical complexity, instrument separation, iatrogenic perforation, cracks, and endodontic-periodontal lesions. The patients were divided into “short-term” and “long-term” groups depending on the time that had passed since the initial treatment (i.e., < 5 and > 10 years, respectively). The causes of failure in the short-term and long-term group were analyzed and compared using logistic regression analyses. Subgroup analysis was performed according to the number of years since the initial treatment in the short-term group to further investigate the association between the time and cause of failure (i.e., < 1, 2, 3, and 4 years, respectively).

**Results:**

Untreated additional canals were present in 21.7% of all cases, and in 36.9 and 6.4% of cases in the short-term and long-term groups, respectively. Multivariable analysis showed that the presence of untreated additional canals was significantly associated with short-term compared to long-term failure. Untreated additional canals were also associated with endodontic failure within 1, 2, 3, and 4 years.

**Conclusions:**

The presence of untreated additional canals was a predictor of endodontic failure within 5 years following initial root canal treatment. To optimize long-term prognosis, it is important to detect and treat all root canals during the initial treatment.

## Background

Root canal treatment (RCT) prevents or treats periapical disease by eliminating microorganisms and blocking their reentry into the root canal system [1, 2]. Successful endodontic treatment requires appropriate chemomechanical preparation and the creation of a tight seal around the root canal with sufficient coronal restoration [3]. The outcome of RCT depends on pre-, intra-, and postoperative factors, such as preoperative periapical status, the apical extent of root filling, and the quality of obturation and coronal restoration [4–10].

Although RCT is a predictable treatment approach with a high success rate, failure occurs in 7–18% of initial RCTs [4, 11, 12]. Endodontic treatment failure (ETF) is defined as the persistence of microbial infection/reinfection in the root canal and/or periradicular lesion [13–16]. Persistent intraradicular infection, which is the most common cause of periradicular disease, results from the bacterial contamination of poorly treated and/or untreated canals, dentinal tubules, root canals with anatomical irregularities, deltas, and isthmus areas. Extraradicular causes of RCT failure, including periapical actinomycosis, foreign body reaction, accumulation of endogenous cholesterol crystals, and true cysts, have also been reported [16–19].

Previous studies have identified the predictors of tooth survival after RCT [4, 7, 11, 12, 20], as well as the causes of ETF [21]. The follow-up duration in studies of survival outcomes after RCT has ranged from 6 months to 30 years [22]. The American Association of Endodontists recommends that clinical and radiographic assessments be conducted for at least 4–5 years following RCT to determine endodontic success [23]. A systematic review found that 86.4, 93.3, and 86.7% of teeth survived for 2–3, 4–5, and 8–10 years after the initial treatment, respectively [12].

Knowledge regarding when and why RCT failure occurs could aid the prediction of treatment outcomes and guide decision-making during retreatment. Furthermore, the potential factors contributing to failure in the short term may differ from those that cause failure over a longer period of time. Yet, few studies have investigated the causes of ETF according to the time since the initial treatment. This is likely because of frequent uncertainty regarding the time of completion of the initial treatment, as these treatments are often administered by other practitioners [24, 25]. Understanding factors related to the amount of time that has passed between treatment failure and the initial treatment (i.e., short-term versus long-term) may improve the outcomes of both primary RCT and retreatment. To address this in the present study, we investigated the possible predictors that could differentiate early from long-term RCT failure. Accordingly, we investigated the underlying factors contributing to ETF, as well as the potential correlations between these causes and the duration between the initial treatment and subsequent retreatment. We hypothesized that the time-dependent failure occurrence may vary based on treatment-related factors.

## Methods

The local ethics committee approved the study protocol (no.: ECT 11–34–01). The data were anonymized prior to the analysis to prevent bias and protect the personal information of the participants. The study conformed to the STROBE guidelines [26].

### Study design and participants

This retrospective cohort study enrolled data from 1262 consecutive patients who received endodontic treatment (871 cases of initial RCT and 391 cases of re-RCT) at the Department of Conservative Dentistry, Ewha Womans University Seoul Hospital, Seoul between May 2015 and May 2020. We did not enroll cases with teeth that had experienced ETF (hereafter, ETF teeth) that required extraction or surgical retreatment, or cases with persistent symptoms caused by non-endodontic factors, including concomitant periodontal disease, occlusal trauma, and non-odontogenic pain. All enrolled patients had been reviewed by two faculty members via clinical and radiographic assessments. We included patients aged > 19 years who had permanent teeth that had undergone nonsurgical endodontic retreatment at our department. The retreatment procedures were performed by the same two faculty members.

The exclusion criteria were antibiotic use for ≥3 weeks; analgesic use within 12 h before treatment; concomitant disease (e.g., diabetes, hypertension, cardiac or pulmonary disease, or neurological or psychiatric disorder); pregnancy; cases with primary root canal treatment;

cases with no clinical records regarding the initial RCT date; traumatized teeth; teeth with open apices; transplanted teeth; endodontically treated teeth with a crown fracture without symptoms and/or periapical lesions; endodontically treated teeth with an old crown and/or an old restoration that had fallen out, necessitating a crown replacement, but no symptoms and/or periapical lesions; endodontically treated teeth with secondary caries but no symptoms and/or periapical lesions.

**Fig. 1:**
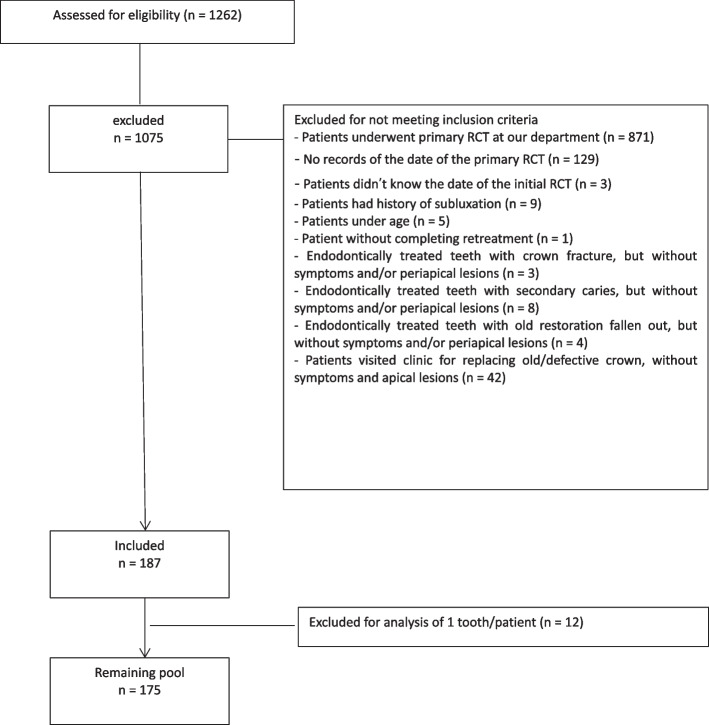
Flow chart showing the patient selection process

One thousand seventy-five cases were excluded according to one or more of the exclusion criteria (Fig. 1). We also excluded 12 teeth to ensure that only 1 tooth per patient was used in the analysis. Therefore, 175 patients (175 teeth) were included in the analyses.

### Clinical and radiographic assessment

The electronic records were independently reviewed twice by two endodontists to obtain information regarding reasons for the visit, demographic characteristics, preoperative periapical and coronal status, intraoperative findings including tooth type (e.g., anterior, premolar, molars) and tooth location (e.g., maxillary, mandibular), and the date of the primary RCT or the time that had elapsed since the initial treatment (Table 1). The patients provided self-reported information regarding the length of time that had elapsed because their initial treatment. For patients who were referred to our center, the referral letter and previous clinical records were examined to corroborate this information. Periapical radiographs were independently evaluated by two members using a PACS viewer (INFINITT Healthcare, Seoul, Korea) with a high-resolution 27-in. monitor (SE2717H; Dell, Round Rock, TX). Any disagreements between the examiners were resolved through discussion.

**Table 1:** Demographic and clinical characteristics of the patients (*n* = 175)

Variables	Categories	*n*
Age	Mean (SD) in years	48.75 (16.1)
	< 35 years	33
	35 ≤ < 60 years	100
	≥ 60 years	42
Sex	Female	99
	Male	76
Tooth type	Anterior	30
	Premolar	31
	Posterior	114
Tooth location	Maxillary	91
	Mandibular	84
Periapical diagnosis	Symptomatic apical periodontitis (SAP)	81
	Asymptomatic apical periodontitis (AAP)	30
	Acute apical abscess (AAA)	12
	Chronic apical abscess (CAA)	50
Periapical radiolucency	No (PAI score = 1, 2)	36
	Yes (PAI score = 3, 4, 5)	139
Preoperative pain	None	41
	Pain or discomfort on biting/tenderness to percussion	69
	Pain triggered spontaneously	26
	Both spontaneous pain and pain on biting	37
	Pain or discomfort triggered by thermal stimulation (by hot food)	2
Type of coronal restoration	Resin	27
	Amalgam	7
	Crown	140
	Temporary filling state	1
Time that has passed since the initial treatment (years)	< 5	65
	5 ≤ < 10	31
	10 ≤	79
Reasons for visit	Pain or discomfort on biting/tenderness to percussion	39
	Spontaneous pain	14
	Both spontaneous pain and pain on biting	26
	Persistent pain or discomfort on percussion after primary RCT	20
	Sinus tract	47
	Gingival swelling	11
	Other reasons	18

Other reasons for visit included an increase in the size of the periapical lesion (*n* = 1), a defective restoration with or without secondary caries (*n* = 9), secondary caries (*n* = 6), crown fracture (*n* = 1), and an old crown that had fallen out (*n* = 1)

Only one reason for the visit was noted

Teeth with endodontic treatment failure were defined as those with periapical lesions that required endodontic retreatment or that caused symptoms such as spontaneous pain, pain triggered by biting and/or tenderness on percussion, swelling, sinus tract, and so forth. The potential causes of failure considered in this study included inadequate obturation quality, inadequate coronal status, the presence of an untreated additional canal, anatomical complexity (e.g., a C-shaped, isthmus, or anomalous root canal), instrument separation, iatrogenic perforation, the presence of a crack, and concomitant endodontic-periodontal lesion.

Obturation quality was assessed via periapical radiographs and classified according to the length and homogeneity of the root filling, as described by Tronstad et al. [27]. The radiographic quality of the root canal fillings was categorized as follows:

1. Adequate obturation quality: The filling material ended 0–2 mm from the radiographic apex and all canals were homogenously obturated with no voids.
2. Inadequate obturation quality: The root filling terminated > 2 mm from the radiographic apex or extended beyond the radiographic apex (underfilled and overfilled, respectively) and/or was inhomogeneous with voids within the filling material or a gap between the root filling and dentin.

Coronal status was assessed clinically and radiographically. The type and quality of coronal restoration, along with signs of coronal leakage, were evaluated based on electronic databases and radiographs. In accordance with the modified criteria described by Tronstad et al. [27] and Hommez et al. [5], coronal status was categorized as follows:

1. Adequate coronal status: Permanent restoration with intact margins and no signs of coronal leakage or caries.
2. Inadequate coronal status: Permanent restoration with evidence of recurrent caries, open margins, overhangs, fractures, or cracks, or provisional restoration with coronal leakage.

We clinically and radiographically evaluated the presence of additional untreated or missed canals, with or without calcified orifices other than the main canal, such as the second mesiobuccal canal of the maxillary molars, distolingual, or middle mesial canal of the mandibular molars, and additional canals of the anterior, premolar, molar, or any C-shaped canal system. Untreated additional canals were distinguished from untreated calcified canals, which typically could not be treated up to the apex because of calcification. These intraoral examination findings were obtained from the clinical records and then verified by periapical radiographs. The presence of C-shaped canal was confirmed from the electronic charts.

The cases were divided into two groups based on the time since the initial treatment: 1) the “short-term” group, for whom less than 5 years had passed since the initial treatment; and 2) the “long-term” group, for whom more than 10 years has passed since the initial treatment. After identifying the predictors for endodontic failure within 5 years compared to those for long-term failure, the short-term group was subdivided into the 4, 3, 2, and 1 year(s) groups to further assess the associations between specific predictors and short-term failures.

### Statistical analyses

Statistical analyses were performed using SPSS software (version 20.0; IBM Corp., Armonk, NY). The chi-square test was used to evaluate the differences in baseline characteristics between the groups. Univariable and multivariable logistic regression analyses were performed to determine the predictors of endodontic failure within 5 years after the primary RCT. To evaluate further the associations between the predictors of endodontic failure and the time interval since the initial treatment, multivariable models were used to predict the dependent variable (i.e., time interval; 1–4 years) based on the independent variables (i.e., predictors of endodontic failure).

## Results

**Fig. 2:**
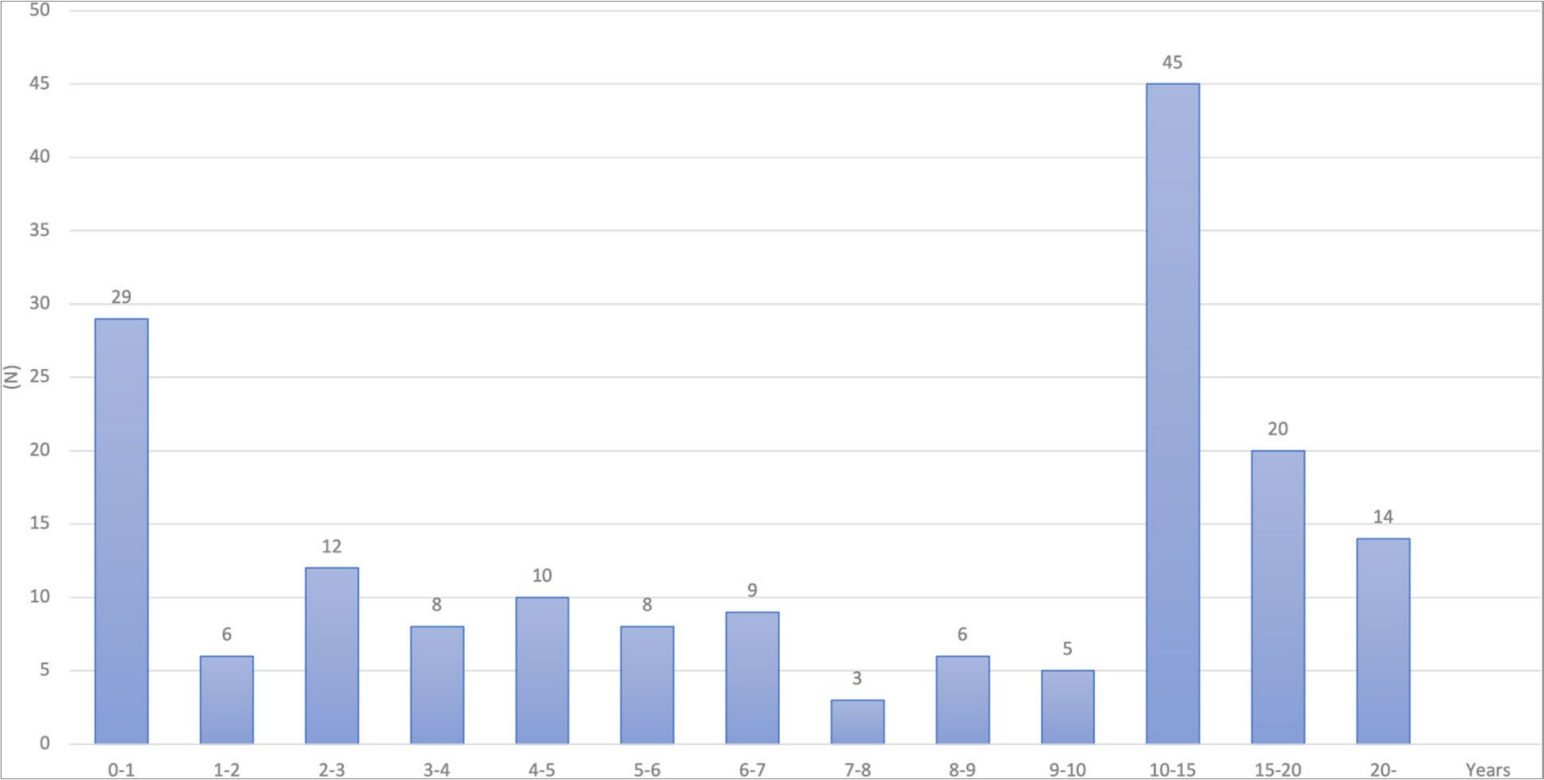
Time interval between the initial treatment and treatment failure

The most frequent reason for seeking endodontic retreatment at our department was sinus tract formation (26.9%), followed by pain on biting and/or tenderness on percussion (22.3%; Table 1). The mean length of time between the initial RCT and additional treatment was 8.6 ± 7.1 years. Of the 175 patients, 45% required retreatment more than 10 years after the initial treatment, while 37% required retreatment within 5 years (Table 1; Fig. 2). The chi-square test was used to evaluate differences in baseline characteristics between the short-term and long-term groups. No significant differences were observed between the groups in terms of age (*p* = 0.051), sex (*p* = 0.403), tooth location (*p* = 0.276), apical diagnosis (*p* = 0.111), or preoperative pain (*p* = 0.874). However, there was a significant difference between the groups in terms of tooth type (*p* = 0.026).

### Causes of ETF teeth

**Fig. 3:**
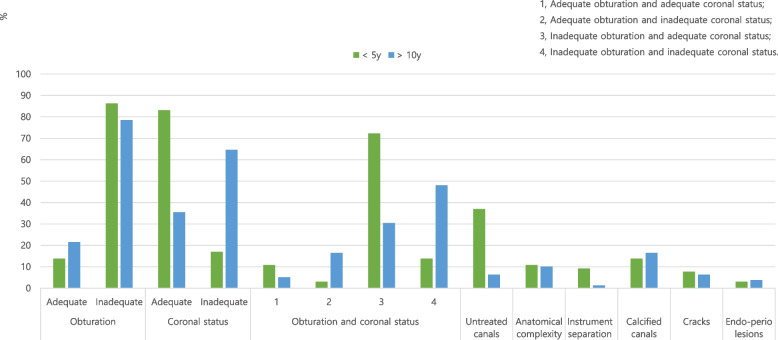
Potential causes for failure of endodontically treated teeth in the short-term (< 5 years) and long-term (> 10 years) groups

The potential causes of RCT failure (i.e., obturation quality, coronal status, presence of an untreated additional canal, anatomical complexity, instrument separation, iatrogenic perforation, presence of a crack, and concomitant endodontic-periodontal [endo-perio] lesion) in the short-term and long-term groups are shown in Fig. 3. Obturation quality was inadequate in 86.2% of the short-term group and 78.5% of the long-term group, with no significant difference between groups (*p* > 0.05). Coronal status was adequate in 83.1% of the short-term group, whereas 35.4% of the individuals in the long-term group had adequate coronal status (*p* < 0.05). Additional untreated canals were present in 36.9 and 6.3% of cases in the short-term and long-term groups, respectively (*p* < 0.05). The distribution of other factors (i.e., complex anatomy, instrument separation, iatrogenic perforation, presence of a crack, and endo-perio lesions) was not significantly different between the two groups (*p* > 0.05).

### Predictive factors for endodontic failure within 5 years

Logistic regression analyses were performed to determine the predictors of failed endodontic treatment within 5 years. The associations between the endodontic treatment failure cause and time (i.e., failure within 5 years or more than 10 years) are shown in Table 2. The involvement of molars and the presence of untreated additional canals were significantly associated with an increased likelihood of endodontic failure within 5 years. Age and inadequate coronal status were associated with a significantly reduced likelihood of endodontic failure within the first 5 years.

**Table 2:** Univariable logistic regression analysis to identify predictive factors for endodontic failure within 5 years compared to long-term endodontic failure

				Single logistic regression analyses			
					95% CI		
		< 5y (*n* = 65)	> 10y (*n* = 79)	Odds ratio	Lower	Upper	*P*
Patient factor	Age (per year)	45.02 ± 15.15	51.77 ± 16.82	.975	.955	.997	.023*
	Sex						
	Male	30 (46.2)	31 (39.2)	1 (reference)			
	Female	35 (53.8)	48 (60.8)	.757	.387	1.480	.416
Tooth factor	Tooth type						
	Non-molar	16 (24.6)	33 (41.8)	1 (reference)			
	Molar	49 (75.4)	46 (58.2)	2.296	1.104	4.774	.026*
	Tooth location						
	Mandibular	27 (41.5)	40 (50.6)	1 (reference)			
	Maxillary	38 (58.5)	39 (49.4)	1.460	.748	2.846	.267
	Complex anatomy						
	Non-C-shaped	58 (89.2)	71 (89.9)	1 (reference)			
	C-shaped	7 (10.8)	8 (10.1)	.934	.307	2.847	.905
	Calcified canals (Presence)	9 (13.8)	13 (16.5)	.846	.336	2.129	.723
	Crack (Presence)	5 (7.7)	5 (6.3)	1.276	.352	4.618	.710
	Endo-perio lesion (Presence)	2 (3.1)	3 (3.8)	.409	.041	4.026	.443
Treatment factor	Obturation quality						
	Adequate	9 (13.8)	17 (21.5)	1 (reference)			
	Inadequate	56 (86.2)	62 (78.5)	1.706	.704	4.134	.237
	Coronal status						
	Adequate	54 (83.1)	28 (35.4)	1 (reference)			
	Inadequate	11 (16.9)	51 (64.6)	.112	.050	.248	.000*
	Obturation quality and coronal status						
	Adequate obturation and adequate coronal status	7 (10.8)	4 (5.1)	1 (reference)			
	Adequate obturation and inadequate coronal status	2 (3.1)	13 (16.5)	.088	.013	.606	.014*
	Inadequate obturation and adequate coronal status	47 (72.3)	24 (30.4)	1.119	.298	4.203	.868
	Inadequate obturation and inadequate coronal status	9 (13.8)	38 (48.1)	.135	.032	.564	.006*
	Additional untreated canals (Presence)	24 (36.9)	5 (6.3)	9.108	3.224	25.732	.000*
	Instrument separations (Presence)	6 (9.2)	1 (1.3)	8.211	.962	70.091	.054

*Significant at *P* = .05

The clinical variables that were significant in univariable regression analysis were entered into a multivariable regression model (Table 3). In the multivariable model, the presence of untreated additional canals was correlated with a significantly increased likelihood of endodontic failure within the first 5 years. However, inadequate coronal status, regardless of obturation quality, was associated with a significantly decreased likelihood of endodontic failure within the first 5 years.

**Table 3:** Multivariable logistic regression analysis to identify predictive factors for endodontic failure within 5 years

Explanatory variables (test category/reference category)	Endodontic failure within 5 years			
		95% CI		
	Odds ratio	Lower	Upper	*P* value
Age (per year)	.978	.953	1.003	.079
Sex (female/male)	.489	.205	1.169	.108
Tooth type (molar/non-molar)	1.385	.541	3.549	.497
Complex anatomy (C-shaped/non-C-shaped)	1.457	.374	5.671	.588
Obturation quality (inadequate/adequate)	1.177	.413	3.356	.760
Coronal status (inadequate/adequate)	.121	.048	.305	.000*
Separated instrument (presence/absence)	8.406	.701	100.773	.093
Untreated additional canals (presence/absence)	3.770	1.108	12.828	.034*

*Significant at *P* = .05

A multivariable model was used to determine the association between the presence of untreated additional canals and specific failure time intervals (i.e., 1–4 years). After adjusting for other variables, the presence of untreated additional canals was significantly correlated with short-term endodontic failure, i.e., failure after < 1 year and < 2 years (Table 4).

**Table 4:** Multivariable logistic regression analysis showing the association between the presence of untreated additional canals and short-term endodontic failure, adjusted for obturation quality and coronal status

	Untreated additional canals		
	Presence	Absence	
	Odds ratios (95% CI)		*P* value
Adjusted for obturation quality, coronal status, and time < 5 years	4.512 (1.5–13.571)	1 (reference)	.007*
Adjusted for obturation quality, coronal status, and time < 4 years	5.428 (1.721–17.124)	1 (reference)	.004*
Adjusted for obturation quality, coronal status, and time < 3 years	6.210 (1.836–21.007)	1 (reference)	.003*
Adjusted for obturation quality, coronal status, and time < 2 years	5.404 (1.453–20.103)	1 (reference)	.012*
Adjusted for obturation quality, coronal status, and time < 1 year	6.070 (1.474–24.992)	1 (reference)	.013*

*Significant at *P* = .05

## Discussion

Determining the causes of failure in endodontic treatment is the first and most important step in decision-making regarding retreatment [3, 28]. Clinicians are expected to have thorough knowledge of the possible causes of endodontic failures, such as inadequate root filling, coronal leakage, iatrogenic problems, and untreated (missed) canals [29]. Previous studies have investigated the clinical factors and conditions that influence the outcomes of primary RCT [4, 11, 12, 20, 30], as well as the predictors of endodontic failure [21]. Endodontic failure occurs at different time points after the primary RCT, and the reasons for failure may differ between short- and long-term failures. However, to the best of our knowledge, no previous studies have evaluated the relationships between the timing and causes of endodontic failure following primary RCT. In the present study, we explored potential predictors of treatment failure within 5 years and more than 10 years after the initial treatment to support more accurate long-term prognosis.

In a previous study [10], inadequate root fillings were observed in 78.6% of endodontically treated teeth with diseased apical status, while inadequate coronal restorations were found in 41.8%. In line with earlier research, this study found that 81.9 and 43.1% of cases had an inadequate root filling and inadequate coronal status, respectively. This study included ETF teeth with symptoms and/or periapical lesions that required retreatment. Most ETF teeth showed poor root fillings with no significant difference between the short-term and long-term failure groups, indicating that poor RCT may lead to treatment failure regardless of the time since the initial treatment. This is likely to negatively affect both short- and long-term tooth survival.

The quality of a root filling and coronal restoration are prognostic factors affecting the endodontic treatment outcome [4, 31]. Ray and Trope reported that when using radiographic assessments alone, the quality of coronal restoration was more strongly associated with endodontic success than the quality of the root filling [30]. This demonstrates the importance of coronal status in endodontic outcomes. Although an adequate coronal seal is essential for successful endodontic treatment, previous studies using radiographic and clinical assessments have found that coronal restoration had a minor impact on endodontic success [32, 33]. Other studies have found that preexisting apical periodontitis and the quality of the root canal filling significantly affect the long-term outcomes of RCT [6, 7, 20]. According to a systematic review [31] and several other studies [5, 8–10], adequate root canal filling and coronal restoration are correlated with endodontic success. In the present study, the rate of ETF teeth with inadequate obturation quality was not significantly different between the groups, whereas the rate of inadequate coronal status was significantly lower in the short-term group compared to the long-term group (16.9% vs. 64.6%). Most teeth with endodontic failure within 5 years had adequate coronal status, regardless of the obturation quality. Because our study was conducted at a university medical center, a large proportion of our patients were referred from local clinics for treatment of endodontic failure or persistent pain/discomfort after an initial treatment, followed by coronal restoration. The characteristics of the included patients may explain the low rate of inadequate coronal status in ETF teeth within the first 5 years.

In this study, the overall incidence of untreated additional canals was 20.1%. A previous study [28] reported missed canals in 19.7% of ETF teeth upon microscopic inspection during microsurgery. The authors suggested that the most common cause of endodontic failure was a leaky canal, which led to leakage or a gap between the root filling material and dentin, followed by a missed canal and underfilling. Another study [34] reported missed canals in 23% of healthy and diseased endodontically treated teeth; the most frequently missed canal was the mesiobuccal second canal in the maxillary first molars. The authors reported a strong correlation between the presence of a missed canal and apical disease. A previous study [35] revealed that endodontic treatment failure was not related to inadequate filling but rather to inadequate cleaning and shaping, and emphasized the importance of eliminating microorganisms from treated root canals. In the present study, there was no significant difference in the rate of inadequate obturation between the two groups; however, there was a significantly greater proportion of untreated additional canals in the short-term failure group than in the long-term failure group (36.9% vs. 6.3%). Given that a missed canal is frequently associated with short-term endodontic failure, locating it during the initial treatment can help improve the long-term prognosis. Based on our results, we suggest that cases that require early endodontic retreatment be evaluated for the presence of untreated canals to eliminate any remaining microorganisms. Therefore, in cases requiring early retreatment, ultrasonic assessments and microscopic examination according to root canal anatomy could be essential to treatment success. In addition, limited field-of-view cone-beam computed tomography is recommended to localize the missed canals for nonsurgical retreatment, as suggested by a previous study [34] and the American Association of Endodontists and American Academy of Oral and Maxillofacial Radiology joint statement [23].

In this study, age, tooth type, and complex canal anatomy (e.g., C-shaped canal) did not significantly affect the likelihood of endodontic failure within 5 years. C-shaped canals are observed in 40% of the mandibular second molars in East-Asian populations [36]. In the present study, C-shaped canals in the mandibular second molars were present in 54.5 and 41.7% of the short-term and long-term failure groups, respectively, and there were no significant differences in the proportion of cases with C-shaped canals between the groups. A previous study showed that the presence of a C-shaped canal in the mandibular second molar did not negatively affect treatment outcomes [37]. A systematic review [4] found that age, tooth type, and anatomical complexity did not negatively impact the treatment success rate, although associated apical infection had a significant effect on the outcomes. Regardless of tooth type and the complexity of the root canal morphology, adequate infection control and prevention are crucial for successful RCT.

The main limitation of the present study was that we could not determine the pre-, intra-, and postoperative conditions at the time of the primary RCT, such as the presence of preoperative apical periodontitis, size of the periapical lesion, pulp status, use of rubber dam isolation, size of the apical preparation, type of irrigant used, use of medicament, number of treatments, qualifications of operators, or time interval between the completion of RCT and placement of the permanent restoration. The outcome of primary RCT is affected by clinical heterogeneities including pre-, intra-, and postoperative factors, periodontal conditions, and patient characteristics [4, 6, 7, 20]. However, a systematic review reported that the outcomes of endodontic treatment were not consistently associated with pre- and intraoperative factors [25]. Another systematic review reported that most intraoperative factors could not be assessed because of insufficient data [4]. In the present study, 12 causative factors were evaluated: sex, age, tooth type and location, anatomical complexity, calcified canals, cracks, endo-perio lesions, obturation quality, coronal status, untreated additional canals, and instrument separation. Although we were able to collect data related to these factors, further studies are required to identify outcome predictors with respect to clinical variability in the initial treatment.

Previous studies of RCT outcomes have assessed the quality of root filling and coronal restoration using radiographic assessments [27, 30] or both radiographic and clinical assessments [9, 32]. One of those studies found only moderate agreement between radiographic and clinical assessments of coronal restoration, indicating that these assessments should be used together for optimal results [32]. In another study [10] and the present one, the intraoral examination findings were extracted from the clinical records to determine the quality of the coronal restorations. According to the Oxford Centre for Evidence-Based Medicine, a retrospective chart review functions as level 3 evidence [38]. Therefore, the strength of the evidence may be improved by performing chairside intraoral inspections in a prospective study.

## Conclusions

Despite the retrospective nature and clinical heterogeneity of our data and limited potential causative factors, our analyses indicate that obturation quality was inadequate in most ETF teeth, regardless of the time since initial treatment. However, untreated additional canals were more prevalent in cases of endodontic failure that occurred within 5 years than in cases of long-term endodontic failure. Therefore, the presence of a missed canal is predictive of early endodontic failure (i.e., within 5 years) and can aid evaluations of overall long-term prognosis.

## Data Availability

No datasets were generated or analysed during the current study.

## Abbreviations

RCT: Root canal treatment
ETF: Endodontic treatment failure
